# Supplementary material for: Stroma secreted IL6 selects for “stem-like” population and alters pancreatic tumor microenvironment by reprogramming metabolic pathways
Source: Cell Death Dis. 2020 Nov 11;11(11):967. doi: 10.1038/s41419-020-03168-4 (PMC7658205; doi:10.1038/s41419-020-03168-4)
Supplement: Supplementary file 1 — Supplementary Figure legends [file 41419_2020_3168_MOESM1_ESM.docx]

**Supplementary Figure Legends**

**Supplementary Figure 1**: Treatment with TNF-alpha did not alter glucose uptake (as measured by 2-NBDG staining) in MIA-PACA2 or SU8686 staining (A). Validation of IL6R silencing in pancreatic cancer cell Su86.86 (B). 2NBDG assay in MIA-PACA2 cells (C).

**Supplementary Figure 2**: Treatment of pancreatic cancer cells (BxPC3 and CFPAC) with IL6 increased their resistance to Gemcitabine induced apoptosis.

**Supplementary Figure 3**: Activation of STAT3 following treatment with PSC conditioned media on MIA-PACA2 (A). Densitometric quantitation of western blot showing pSTAT3 and total STAT3 after IL6 treatment (B) and treatment with PSC conditioned media (C). Inhibition of STAT3 signaling by Stattic decreased IL6 induced CD133+ population (D) and lactate production (E). PDH1 activity assay (kinetics and rate of activity) in MIA-PACA2 cells following IL6 treatment, blocking IL6 signaling by anti IL6R antibody and static respectively (F).

**Supplementary Figure 4**. Proliferation of MIAPACA2 cells were increased upon treatment with IL6 (A) and PSC conditioned media (B). Quantitation of infiltrated CD8+ T cells in IL6 neutralizing antibody treated tumors (C) and WBI-5111 treated tumor (D). Target genes of STAT3 as observed in the CHEA transcription factor target database (E).
