# Supplementary figures and images for: Stroma secreted IL6 selects for “stem-like” population and alters pancreatic tumor microenvironment by reprogramming metabolic pathways
Source: Cell Death Dis. 2020 Nov 11;11(11):967. doi: 10.1038/s41419-020-03168-4 (PMC7658205; doi:10.1038/s41419-020-03168-4)

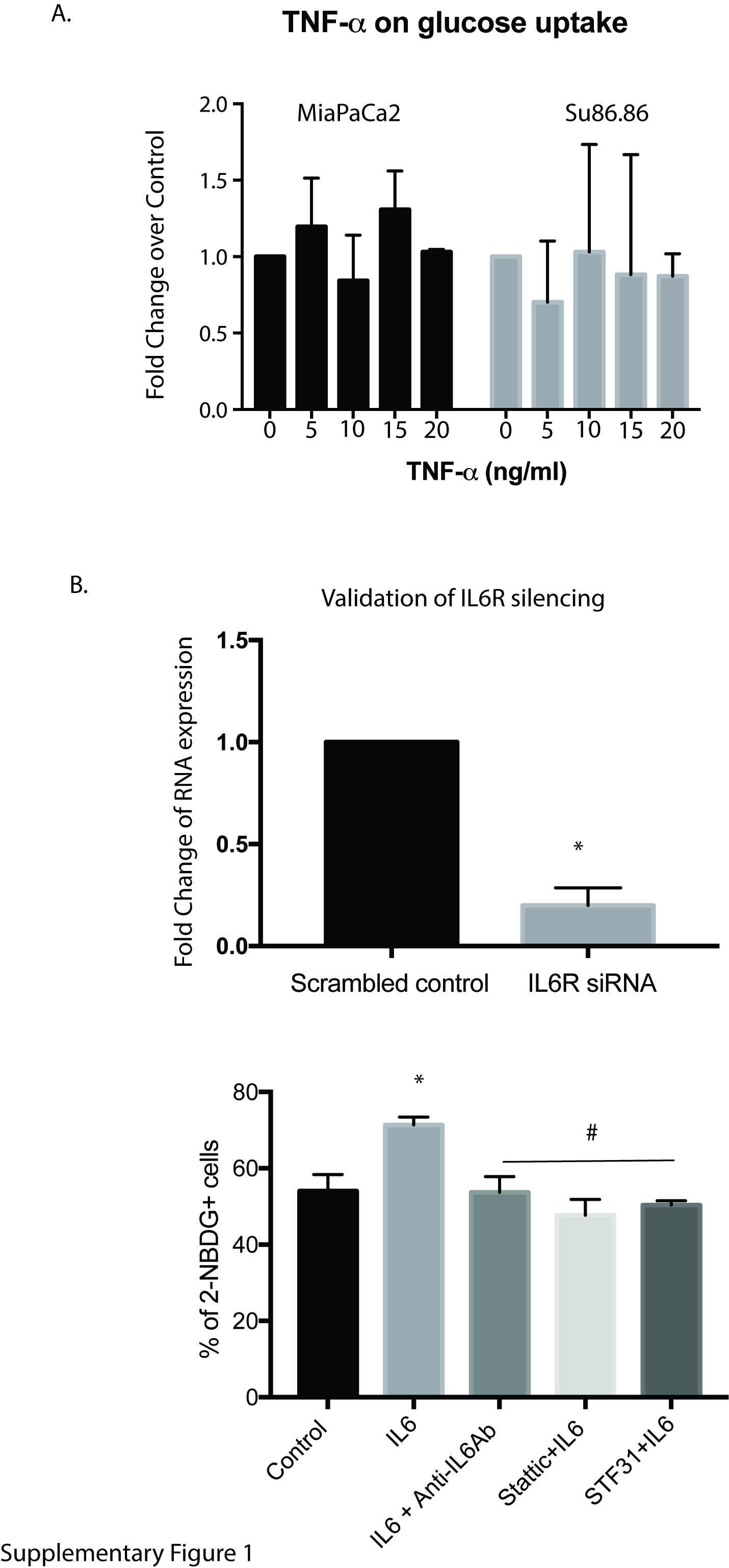

Supplement: Supplementary file 2 — Supplementary Figure 1 [file 41419_2020_3168_MOESM2_ESM.tif]

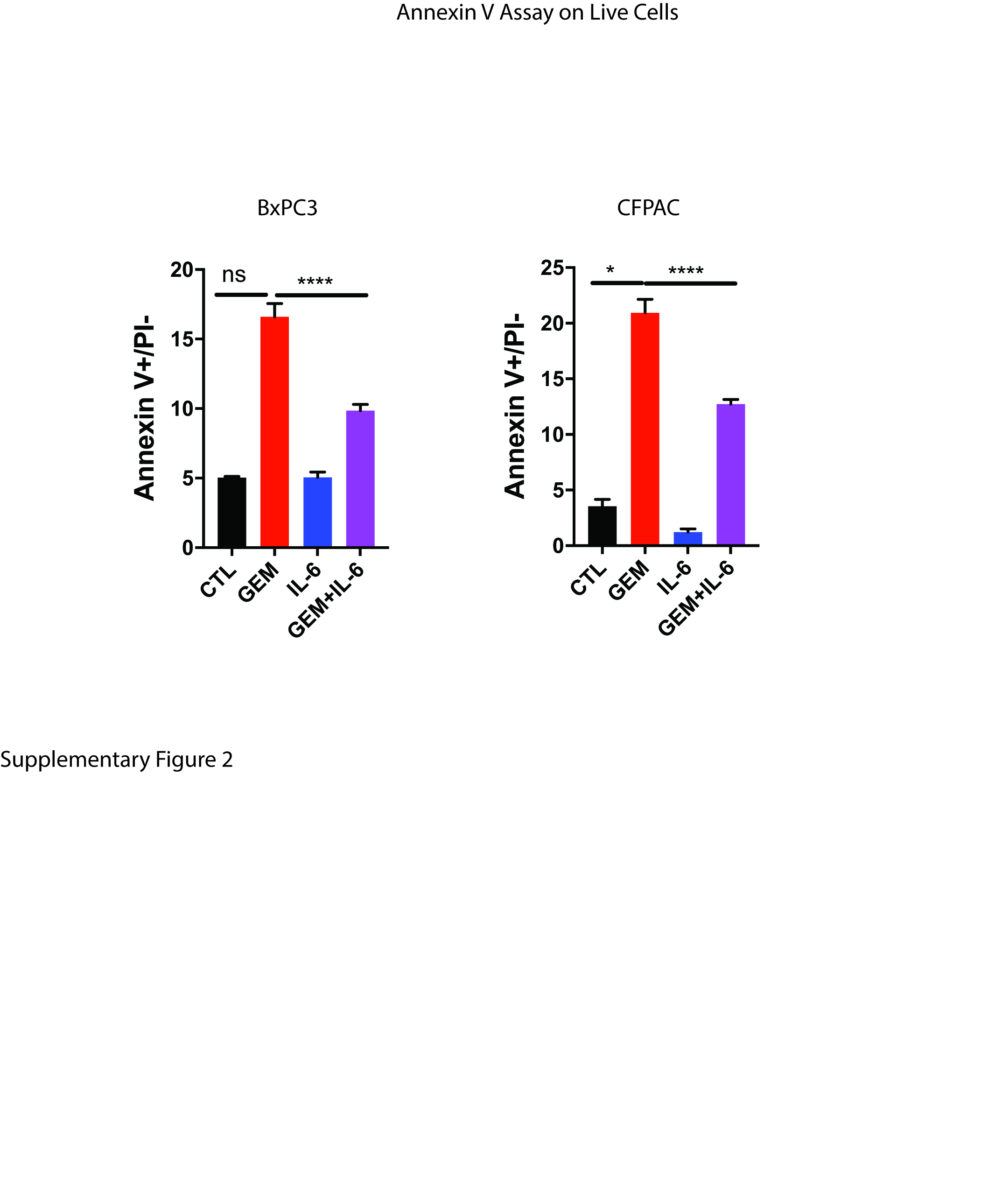

Supplement: Supplementary file 3 — Supplementary Figure 2 [file 41419_2020_3168_MOESM3_ESM.tif]

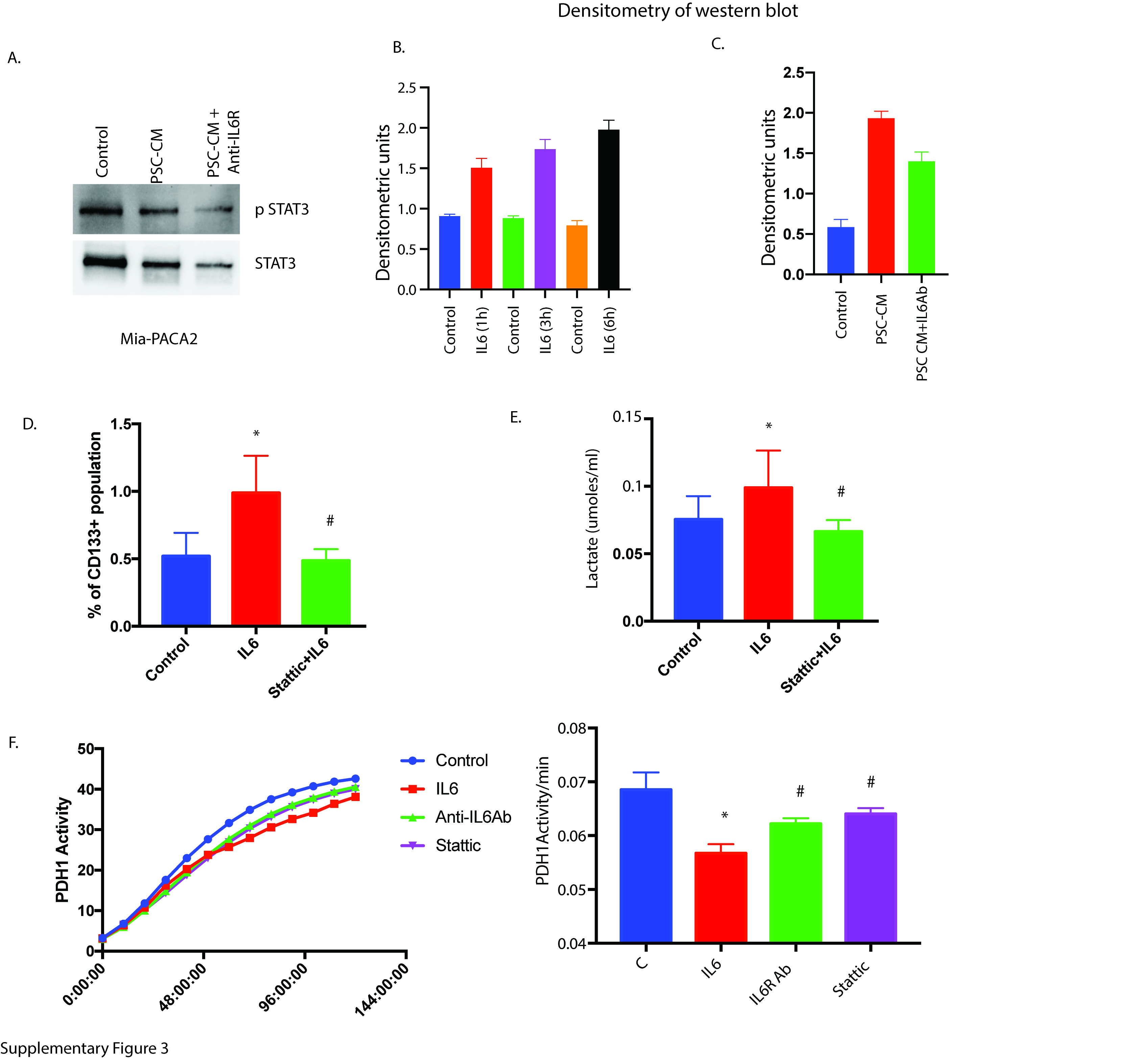

Supplement: Supplementary file 4 — Supplementary Figure 3 [file 41419_2020_3168_MOESM4_ESM.tif]

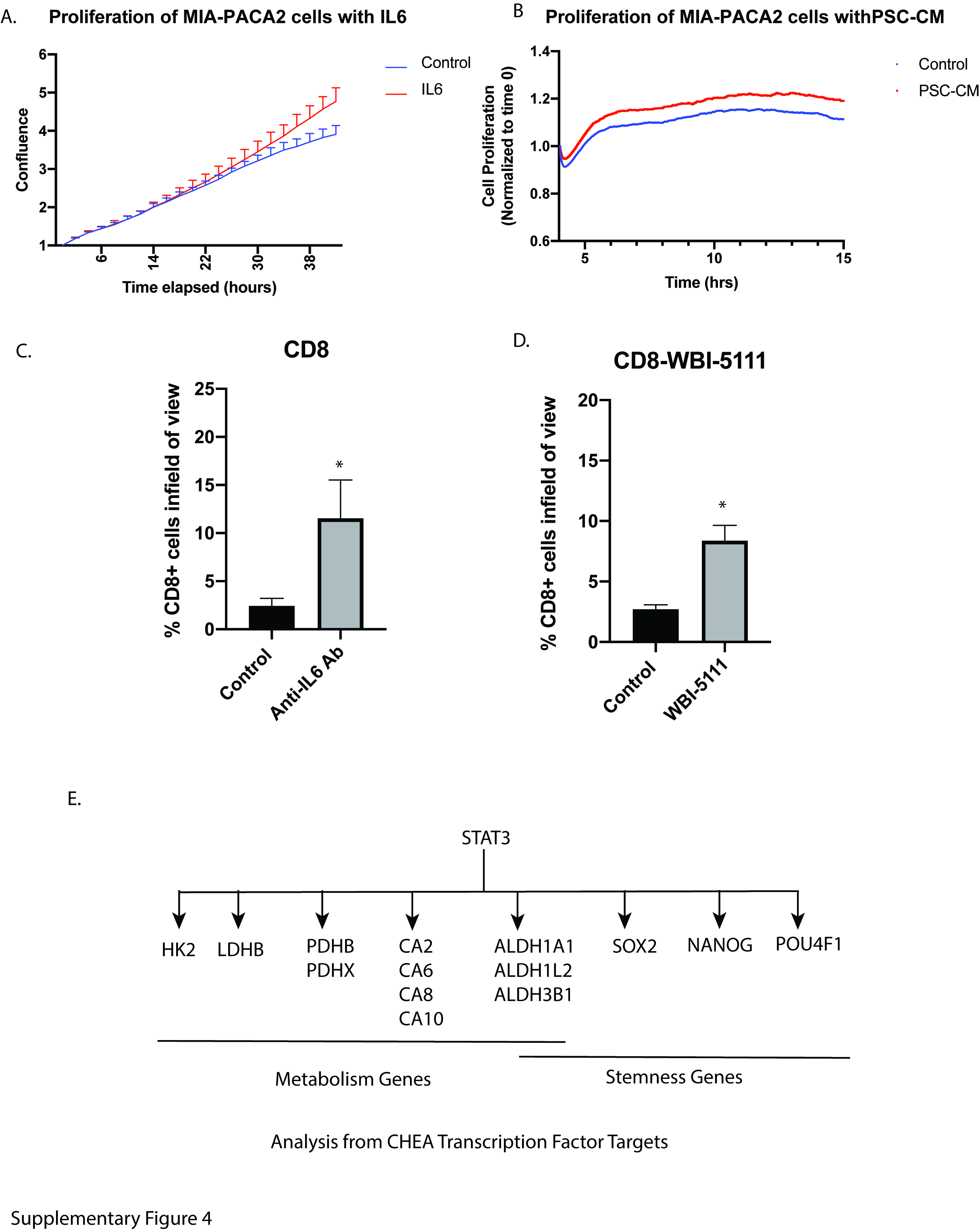

Supplement: Supplementary file 5 — Supplementary Figure 4 [file 41419_2020_3168_MOESM5_ESM.tif]
